# Pilot Outreach Program in Remedis—The Promising Step toward HCV Elimination among People Who Inject Drugs

**DOI:** 10.3390/ijerph20010501

**Published:** 2022-12-28

**Authors:** Laura Krekulová, Tomáš Damajka, Zuzana Krumphanslová, Vratislav Řehák

**Affiliations:** 1 Remedis, s.r.o., Vladimírova 10, 140 00 Prague 4, Czech Republic; 2 4th Department of Internal Medicine, First Faculty of Medicine, Charles University in Prague, Kateřinská 32, 128 00 Prague 2, Czech Republic

**Keywords:** viral hepatitis C, HCV infection, people who inject drugs (PWIDs), WHO HCV elimination plan, off-site service, Comprehensive Care Program (CCP), outreach program Remedis, Prague

## Abstract

The global effort to eliminate HCV infection requires new approaches to accessing and testing the affected population in a setting with as low of a threshold as possible. The focus should be on socially marginalized people who inject drugs (PWIDs) and who are not willing or able to visit standard medical services. With this vision, we established an outreach service—a testing point in an ambulance in the park in front of the Main Railway Station of the capital city of Prague—to provide bloodborne disease testing and treatment. The service was available every week on Wednesday afternoon. Over the initial two years of our experience, 168 unique people were tested. Of them, 82 (49%) were diagnosed with chronic HCV infection and were eligible for treatment with antivirals. Of these, 24 (29%) initiated antiviral treatment over the study period, and 17 (71%) of these individuals achieved a documented sustained virological response. Offering medical services in PWIDs’ neighborhoods helps overcome barriers and increase the chances that they will become patients and begin HCV treatment. The described outcomes appear promising for reaching the vision of linkage to the care of such a hard-to-reach population and can serve as a feasible model of care for further expansion.

## 1. Introduction

Providing care for patients with diagnoses of addiction and other comorbidities is complicated and often requires close collaboration between specialists from different, unrelated fields [1]. A specific risk in people who inject drugs (PWIDs) is the spread of blood-borne infections and the need for their diagnosis and treatment beyond addiction care [2,3]. People who inject psychoactive substances are the main source of new infection outbreaks caused by the hepatitis C virus (HCV) in developed countries [4,5,6].

### 1.1. WHO Plan to Eliminate HCV Infection Worldwide

Viral hepatitis C is a serious chronic disease that has been classified by the WHO as a general threat [7]. An action plan aimed at the global elimination of HCV infection has been in place since 2016 [8,9]. After the implementation of preventive measures (screening of blood donors, using only disposable injection sets in facilities, etc.), chronic viral hepatitis C began to spread, especially among PWIDs [10,11]. The WHO’s action plan includes targeted care for PWIDs, who were identified by the WHO as one of the most high-risk and at-risk groups [8,9,11]. To eliminate HCV infection and accomplish the WHO’s action plan, testing and treatment must be available for all potential patients, including those from excluded communities and socially marginalized PWIDs [12,13,14,15]. “Test and treat” is the only way to bring the HCV epidemic among PWIDs under control and to eliminate HCV outbreaks among them [11,15].

The need for concurrent treatment of addiction and viral hepatitis creates new, often unpredictable, and difficult-to-resolve obstacles and, therefore, requires close collaboration among health professionals from different disciplines. In theory, we are ready to eliminate HCV infection, but there is an urgent need to change our testing and treatment stra-tegies to reach all at-risk patients and link them to the healthcare system

### 1.2. Comprehensive Care Program for Patients with Addiction Comorbidity in Remedis, Prague

Two decades before the presentation of the WHO’s international program announcing the global plan for the elimination of hepatitis C (HCV) infection, the Comprehensive Care Program for patients with addiction comorbidity (hereinafter referred to as “CCP”) was created in Remedis in Prague, Czech Republic, to treat patients with liver disease and co-occurring substance use disorders. In accordance with the present WHO recommendations, the CCP has always emphasized the improvement of access to care, diagnostics, and treatment, as well as the inclusion of individuals and groups from excluded communities in medical care and specific HCV treatment. In the year 2019, the Remedis healthcare facility opened a new outreach service, “Ambulance”, to offer medical care to PWIDs more efficiently, with the goal of offering HCV treatment.

#### 1.2.1. The Development and Basic Principles of the Comprehensive Care Program (CCP) in Remedis

**Figure 1:**
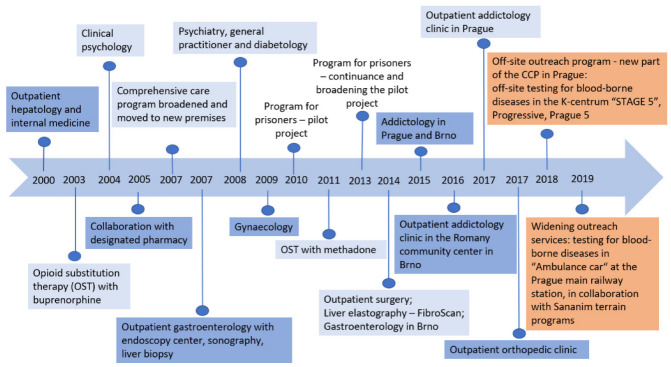
Development of the Comprehensive Care Program for patients with addiction comorbi- dity in the Remedis healthcare facility in Prague and Brno in the Czech Republic [16].

Remedis is a single non-state multidisciplinary medical facility. It was founded in Prague in 2000, and in 2014, a branch was established in Brno. The CCP was created as a specific part of this healthcare facility (the evolution of Remedis and the CCP is summarized in Figure 1). The need to address liver diseases in active drug users led to the creation of this interdisciplinary program. The CCP provides patients with a wide range of services in a single healthcare facility. The main objective is to reduce the somatic, psychological, and social risks associated with the use of illicit drugs. Apart from the treatment of infectious diseases, the goal of the comprehensive approach is to establish a long-term collaboration with patients and to help them with lifestyle changes that prevent relapses into substance use, as well as reinfection with viral hepatitis C. Years of experience have shown that sufficient support, empathy, and respect for PWIDs who are living with chronic viral hepatitis C can foster very good collaboration. The facilitated contact improves the doctor–therapist–patient/client–healthcare facility relationships. The goal is to reduce the somatic, psychological, and social risks associated with the use of illicit drugs. Aside from complete abstinence from drugs (which is a major task, but often unattainable), the most important thing is to ensure the stabilization of patients with addiction.

The CCP was developed over time together with the necessity of solving the problems and needs of patients with addiction and chronic viral hepatitis C (see Figure 1). At that time, the only available treatment for chronic viral hepatitis C was interferon treatment [17,18]. This treatment was covered by health insurance, but it required a number of pre-treatment examinations, such as abdominal ultrasound and liver biopsy. In the beginning, we would send our patients who injected drugs to undergo these examinations at different healthcare facilities. The accessibility of healthcare for these patients proved to be very poor. This was the impetus for establishing a healthcare facility that was tailored to the specific needs and options of PWIDs. For the last 10 years, all patients have been provided with comprehensive outpatient care in the fields of internal medicine, hepatology, gastroenterology, gynecology, diabetology, general practice, X-ray, surgery, psychiatry, clinical psychology, social work, and addictology.

The underlying idea of the CCP is a “low-threshold” healthcare facility that provides care for all patients without any further obstacles (requirements for access to healthcare). We do not require PWIDs to have recommendations from a primary care doctor (which they usually do not have), and we make appointments at Remedis flexibly. If possible, we perform all of the necessary examinations in one day, i.e., during one visit to Remedis. Most of the services that we offer are under one roof. Outpatient services are interconnected, and there is no complicated administration, handing over of documentation, or any other standard procedures that often exclude PWID patients from regular healthcare facilities. The basic principles of the CCP are summarized in Table 1.

#### 1.2.2. Care in the Hepatology Center

**Figure 2:**
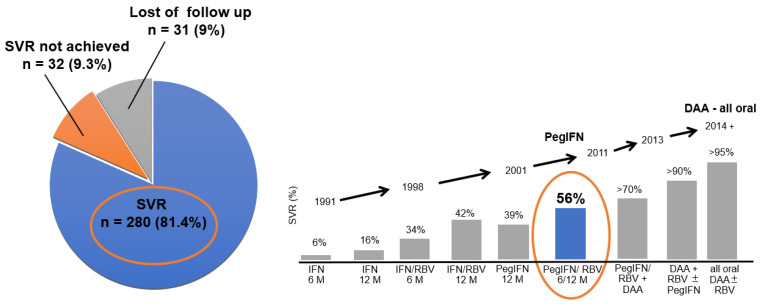
Remedis treatment outcomes for HCV-infected PWID patients treated with pegylated interferons and ribavirin in the years 2005–2010 (SVR 81.4% orange circle) compared with those in registration studies with pegylated interferons and ribavirin realized in the general population—orange circle on bar graph SVR 56% (left side (pie chart)—data from Remedis, Prague; right side (bar graph)—evolution of chronic viral hepatitis C treatment modalities and increase in the efficacy of viral clearance measured as sustained virological response). SVR—sustained virological response: defined as undetectable HCV RNA 24 weeks after treatment completion, IFN—interferon, RBV—ribavirin, PEG-IFN—pegylated interferon, DAA—direct-acting antivirals (adapted from the US Food and Drug Administration’s Antiviral Drugs Advisory Committee Meeting, 27–28 April 2011, Silver Spring, MD, USA) [16,24,25,26].

The cornerstone of the CCP at Remedis is the hepatology clinic, which has been focusing on the diagnosis and treatment of chronic viral hepatitis for many years. An extensive group of patients with chronic viral hepatitis B, C, and D has been diagnosed and treated here. Since 2006, the clinic has had the status of a Center for the Treatment of Viral Hepatitis, as guaranteed by the Czech Society of Hepatology. For several years, it has been the largest hepatology center in the Czech Republic according to the number of patients treated per year. In recent years, in accordance with international and national recommendations for chronic viral hepatitis C treatment, we have been using orally administered direct-acting antivirals (DAAs) that are produced in tablets [19,20,21,22]. We have verified that with the right approach and support for PWIDs, good treatment outcomes can be achieved in this population, which has always been considered as a difficult-to-treat group [7,19,23]. Treatment outcomes (even with the interferon regimens), including the percentage of PWIDs with sustained virological response (SVR), were better in the CCP than treatment outcomes among the general population in registration studies and other projects [24,25]; see Figure 2.

#### 1.2.3. The Latest CCP Service: Off-Site HCV Testing and Treatment in the “Ambulance” Outreach Program

The CCP found that PWIDs accepted the concept of low-threshold specialized healthcare in a clinical setting and further expanded the model to include an outreach component to engage socially vulnerable individuals. In 2018, the CCP at Remedis included new off-site services for PWIDs who were experiencing homelessness. Since June 2019, in collaboration with another nonstate organization, Sananim (namely, with the Sananim terrain programs), a street outreach program offering regular testing for the most common bloodborne and sexually transmitted infections has been in place. The aim of the Remedis outreach initiative is to test as many PWIDs as possible, including socially marginalized individuals who are unlikely to come to a regular healthcare facility or the outpatient CCP in Remedis, Prague. The new medical service is available in an ambulance in the park next to Prague Main Railway Station. For many years, this park has been the center of the illicit drug scene in Prague. Inside the ambulance, Remedis offers professional healthcare services that are run by registered nurses, including blood testing and liver elastography. The outreach service is free of charge, and patients may use it at their own discretion. In an effort to ensure the best possible compliance of and convenience for clients, all of the consultations for results and further recommendations are conducted in the same place, i.e., in the ambulance. If patients are indicated for treatment, they are given an appointment to see a doctor to start this treatment at the CCP Remedis clinic. If necessary, they can be accompanied by a peer or a social worker for this visit. Our objective is to present the results and share our experience with this CCP outreach service, which was evaluated after two years of pilot practice. We describe and present a new model approach to working with PWIDs from socially excluded groups.

## 2. Methods

### 2.1. Study Design, Setting, and Participants

This pilot work was monocentric. The study prospectively recruited clients who vo- luntarily used our services in the park next to Prague Main Railway Station and who decided to get tested because of their risky behavior. All self-identified as active injection drug users (a combination of methamphetamine and other illicit drugs, including opiates) and as either having unstable housing or being homeless. Testing was free of charge for all clients. Everyone who contacted the outreach program was tested, and all agreed to be included in the study. There were no obligations associated with participation in this study, which was probably why all clients agreed to participate and provided and signed their informed consent to process their medical history, demographics, and laboratory data. The data from all study participants who contacted the outreach service during the reporting period were included for the purposes of this study.

Standard testing for bloodborne and sexually transmitted diseases from blood samples, which was performed in the outreach setting by a Remedis nurse, was available once per week on Wednesday afternoons. (Ideally, a total volume of 17.5 mL of blood was drawn from a patient through venipuncture. The samples were transported to a certified laboratory in the evening on the same day and immediately processed.) Each test included pre-test counseling, blood sampling and liver elastography. Patient medical history and epidemiological data were obtained from all subjects in the form of an interview and a structured questionnaire, which were used at Remedis in Prague for several years. The first visit also included setting the date of the client’s check-up with the examination results. During this check-up, the results were discussed with the client and other solutions were offered, including specific virostatic therapy in the case of viral hepatitis.

### 2.2. Laboratory Tests

All subjects were tested for basic serum biochemical parameters (AST, ALT, ALP, GGT, and bilirubin) and contagious diseases: viral hepatitis type A, B, and C, HIV/AIDS, and syphilis. All tests of all collected samples were performed in a certified laboratory using standardized serology tests—commercially available kits—for antibodies. The serology test kits for viral hepatitis A were an Alinity HAVAb IgM, Abbott (Chicago, IL, USA), ALINITY ii analyzer and Alinity HAVAb IgG, Abbott, ALINITY ii analyzer. The initial screening for viral hepatitis B tested for three basic markers: Alinity HBsAg Qualitative II, Abbott, ALINITY ii analyzer, including an HBsAg Confirmatory Test, DiaSorin (Salugia, Italy), LIAISON XL analyzer. The antibodies were Alinity Anti-HBs, Abbott, ALINITY ii analyzer and Alinity Anti-HBc II. An Alinity Anti-HCV, Abbott, ALINITY ii analyzer was used to verify the HCV infection status. For HIV, an Alinity HIV Ag/Ab Combo, Abbott, ALINITY ii analyzer was used; for syphilis, an Alinity Syphilis TP, Abbott, ALINITY ii analyzer and RPR Reagent, Sekure chemistry, Sekisui Medical Co. (Tokyo, Japan), AU 5822 analyzer were used. In the case of positive antigens (HBsAg) or antibodies (anti-HCV), the diagnosis of viral hepatitis B or, rather, viral hepatitis C was further confirmed with virology testing based on direct proof of viral DNA or RNA by using polymerase chain reaction (PCR). Standard diagnostic kits were used to perform diagnostic virology tests. To detect HCV RNA, an Aptima HCV Quant Dx Assay (Hologic, Marlborough, MA, USA), (limit of detection (WHO IS): 7 IU/mL serum, 8 IU/mL plasma) Panther analyzer or HCV Real-TM Quant Dx (Sacace, Como, Italy) was used; for genotyping, HCV Genotype Plus Real-TM (Sacare) was used. When using the Sacace diagnostic kits, the testing was performed on the same RT-PCR cycler Rotor Gene analyzer (Qiagen, Venlo, The Netherlands).

### 2.3. Epidemiological Data and Statistical Analysis

Personal and demographic data were collected during the assessment of the patients’ history and structured interviews. The data were further processed using the Microsoft Excel 365 statistical program and the IBM SPSS Modeler software. To test the normal distribution of variables, a Shapiro–Wilk test was used. To analyze the significance of differences among particular segments of patients, the Fisher exact test and nonparametric tests were used. Specifically, the Mann–Whitney test, Kruskal–Wallis test, and chi-square test were used. All subjects provided and signed informed consent for inclusion in the study prior to participation.

## 3. Results

During the two years of the study period, from 26 June 2019 through 30 June 2021, a total of 168 clients were tested within the outreach service in the park next to Prague Main Railway Station. There were 116 males (69%) and 52 females (31%). The average age of all clients was 38.1 years (SD 7.65); the average age of the males was 38.9 years (SD 7.68), and that of the females was 36.3 (SD 7.67). For 81 clients (48%) (54 males and 27 females: 67% and 33%, respectively), this was the very first contact with a Remedis healthcare facility. The remaining 88 clients (52%) (62 males and 26 females: 70% and 30%, respectively) had previous contact with Remedis in some other way. Of those 88, in 23 cases (26%), the first contact with Remedis happened via the Remedis program for prisoners (another Remedis care activity). Only 65 (39%) of the clients had previous experience with the Remedis healthcare facility in Prague.

Of the entire study group, 133 (79%) clients were anti-HCV positive and 32 (21%) were anti-HCV negative, and in three subjects, anti-HCV antibody testing was not performed. These three clients were not serologically tested because of the small amount of serum sample obtained due difficulties in drawing blood. In these cases, we preferred PCR testing. Of the three clients with unknown serological outcomes, two were negative for PCR HCV RNA, and one was positive, with a low viral load and an inability to detect the HCV genotype. However, the patient with low HCV viremia has not contacted the outreach service or any other Remedis programs since then, so a subsequent blood test was not possible. Among the anti-HCV-positive clients, 93 were males (70%) and 40 were females (30%), with the average age of 38.4 years (SD 7.65) (male: 38.8 years (SD 7.66); female: 37.6 years (SD 7.66)). The complete serological results for the entire study group are summarized in Table 2. A detailed analysis did not detect any statistically significant differences between males and females (Table 2). Higher positivity of anti-HAV IgG, anti-HBc, and anti-HCV was detected among patients who were 35 years and older compared to those who were less than 35 years old. None of the clients tested positive for active syphilis.

**Figure 3:**
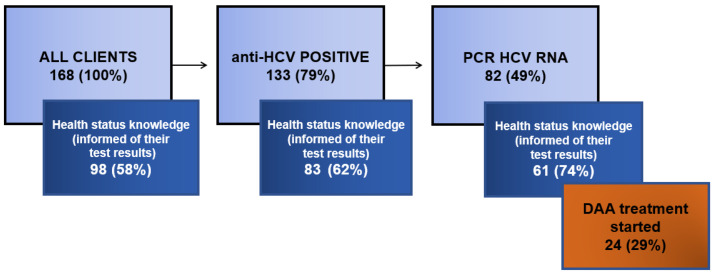
All tested clients in proportion to those who received their test results and were aware of their HCV infection.

Almost half of the study sample who had positive anti-HCV antibodies also had detectable HCV RNA in the serum (n = 82, 49%). Sixty-two percent of all individuals with an anti-HCV-positive result had detectable HCV RNA. The other 51 anti-HCV-positive subjects (38%) were negative for HCV RNA, which means that they were without HCV viremia. Among the anti-HCV-positive patients who were positive for PCR HCV RNA, 56 were males (68%) and 26 were females (32%). The average age of the viremic patients was 38.34 years (SD 7.65) (38.5 for males (SD 7.66) and 38.0 for females (SD 7.66)). Genotypes 1 (subtypes 1a and 1b) and 3 were detected among the PCR-positive subjects from the study group. Of all clients tested in the outreach service, 98 (58%) came for the second (check–up) visit and received their results, as well as the post-test counseling and treatment possibilities, if indicated (Figure 3). Of the 82 HCV-PCR-positive clients, 61 (74%) individuals came for the results and the check-up visit and received the information about their HCV infection. All parameters described above and in Table 2 were tested between the groups of clients who did not come for results and those who came and were informed of their results and treatment possibilities. The tests that were carried out did not confirm any statistically significant differences in this case. 

Of the 82 patients who were diagnosed with chronic viral hepatitis C and were eligible for specific treatment with DAAs, 24 (29%) PWID patients (13 men and 11 women) were able to start treatment; see Table 3. From this particular subgroup of 82 PCR-HCV-RNA-positive clients, there were 38 subjects (46%) (26 men and 12 women—68% and 32%, respectively) for whom the Remedis outreach service represented their first contact with our healthcare facility regarding bloodborne diseases, and 10 (26%) initiated HCV treatment. The average time between the initial contact with the off-site service and the DAA treatment was more than half a year (6.6 months). The average time between the initial contact with the off-site service and the DAA treatment was significantly longer among females and, furthermore, among clients with an age of 40 and more years.

**Figure 4:**
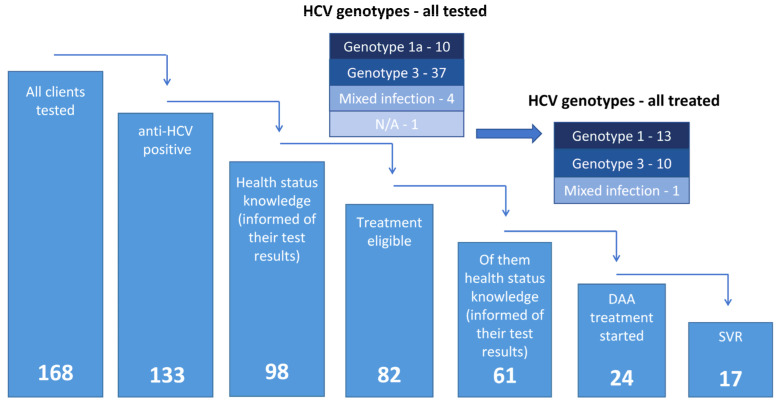
Cascade of care of the Remedis “Ambulance” outreach program. N/A—data not available (genotypes tested with uncertain results); DAA—direct-acting antivirals; SVR—sustained virological response defined as undetectable HCV RNA 12 weeks after treatment completion.

The majority of those treated for chronic viral hepatitis C were treated in our hepatology center in Remedis (17 subjects, 71%). After being diagnosed in the outreach service, five clients (20%) were treated while imprisoned as a part of the Remedis program for prisoners. Two clients (8%) who were initially diagnosed in the outreach program underwent DAA treatments in a different healthcare institution—IKEM Prague (Institute of Clinical and Experimental Medicine, Prague). The treatment details and the treatment outcomes are unknown to us. However, one of these two men appeared in the outreach service again and was diagnosed as being reinfected with different HCV genotyp. HCV genotype 1 was the most common genotype among the treated patients, and it was detected in 13 individuals (54%), followed by genotype 3 (10 cases, 42%). In one subject (4%), coinfection with genotype 1a/3 was diagnosed. Liver elastography was performed in each client, and the mean fibroscan finding of the treated patients was 9.4 (9.7 in males and 9.0 in females), indicating moderately advanced liver fibrosis—for details, see Table 3 and Figure 4. Sixteen PWID patients were treated with fixed DAA combination of glecaprevir/pibrentasvir, which was the preferred treatment regimen because it took the shortest time for treatment. Two patients were treated with grazoprevir/elbasvir, and four patients with advanced liver fibrosis were treated with sofosbuvir/velpatasvir.

Of 22 clients (11 men and 11 women) treated in Remedis, 17 (77%) reached sustained virological response (SVR—sustained virological response defined as undetectable HCV RNA 12 weeks after treatment completion). SVR was documented more frequently among females (due to the incomplete data of the males, it was not possible to test statistical significance), who needed a longer time to initiate the DAA treatment compared to males (as mentioned above). In this sample, females were more likely to complete the full course of treatment and return for testing/documentation of SVR. Fourteen patients returned for testing for curing 12 weeks after completion of DAA therapy. Three patients had their blood drawn after a longer post-treatment time, and they were also negative for PCR HCV RNA. Four patients who underwent HCV treatment did not come for follow-up testing. One other patient is known to have been reinfected with a different genotype during the post-treatment follow-up (FU) period.

## 4. Discussion

The WHO has been calling for the global elimination of viral hepatitis C since 2016 [7,8]. The elimination plan is manageable with modern sensitive diagnostic tools and with new direct-acting antivirals that have been available since 2014 [27,28,29]. One of the limits is the price of the treatment [8]. Involving individuals who are at risk for HCV from high-risk subpopulations, such as PWIDs, can be challenging [30,31]. It can be difficult to engage these individuals in care and treatment not only because of their risky behavior (e.g., injecting illicit drugs, practicing sex work, and tattooing), but also because they are socially excluded due to their low income, homelessness, psychiatric disorders, imprisonment, etc. [32,33]. PWIDs may be receiving suboptimal care due to many barriers, including stigma, housing, criminalization, etc. [34]. In addition, another major barrier to HCV testing of PWIDs is venipuncture, which is recognized by both physicians [35] and patients [36].

At present, PWIDs represent a priority population for targeted scale-up of HCV testing and treatment [5,10,37]. Understanding the factors associated with engagement across the HCV cascade of care (CoC) among PWIDs is essential for developing new targeted interventions. Recent research in Australia (where PWIDs’ involvement in the CoC has been very successful) described the CoC among Australian PWIDs and identified factors associated with involvement at each stage. Accordingly, methamphetamine use (as the drug that was most commonly injected in the last month) was associated with a reduced likelihood of antibodies and HCV RNA testing compared with heroin use. A likely reason for this phenomenon would be that heroin users are usually linked in some way to social and health services through opioid agonist treatment (OAT) [38]. Therefore, additional efforts are needed to reach individuals who are not involved in social services, particularly those who do not receive OAT and who predominantly inject methamphetamine [38]. In addition, PWIDs who reported unstable housing were significantly less likely to initiate HCV treatment [38]. This finding is consistent with those of previous research stating that self-reported homelessness is associated with suboptimal HCV treatment uptake [39,40,41,42,43].

High treatment willingness was documented in the homeless population [44], suggesting that improved treatment accessibility could significantly increase HCV care. However, this requires innovative strategies for engaging people experiencing homelessness in HCV treatment [38,41,42,45]. Flexible service and treatment options have demonstrated benefits for highly marginalized populations, including clients who are currently actively injecting drugs and who do not have stable housing [45]. Targeted outreach services are effective methods for delivering HCV care [46,47]. Integrating and linking HCV testing and treatment with other services that PWIDs attend, including those without stable housing, significantly increases access to HCV treatment for these at-risk individuals [48,49,50,51].

**Figure 5:**
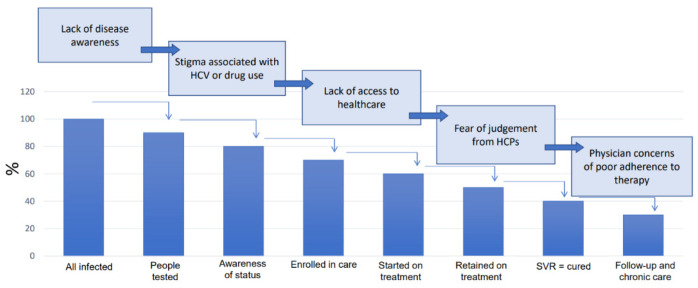
WHO cascade of care. HCP—healthcare professional, SVR—sustained virological response (adapted from: World Health Organization. Global hepatitis report, 2017. Geneva: World Health Organization [7]).

Here, we present our new model for engaging marginalized PWIDs with the health system. The established Remedis medical facility has opened a new type of service that tries to help people “out of the system” to engage in testing for HCV and other infectious diseases and, when appropriate, treatment services. We have linked the outreach service directly with the healthcare facility. This new outreach service and its location (in a park near the Main Railway Station in the current center of Prague drug scene) support individuals who are at risk for HCV in establishing the first contact in the outreach setting, then linking with additional services that are available in healthcare facilities. During the first two years, 168 people received HCV testing and counseling. The anti-HCV seroprevalence of 79% was higher than that estimated in the PWID population in the Czech Republic [52,53], which clearly proves that we involved an endangered subgroup of PWIDs. Of 82 patients who were diagnosed with HCV and eligible for HCV treatment, 24 (29%) were treated with DAAs, and of those who completed treatment, 17 (71%) had documented SVR. Patients with SVR were counted according to the ITT analysis. It is very likely that some of the remaining patients who were lost during follow-up (perhaps all of them) also achieved SVR, but we were unable to document this. The number of patients receiving specific treatment for HCV infection in our pilot study is still not ideal, but is higher than in previously presented conventional CoCs [54,55,56,57]—see Figure 4 and Figure 5. We can all take inspiration from several well-functioning national HCV elimination programs (in Georgia, Australia, Scotland, etc.) [58]. Georgia had the highest percentage of DAA treatment uptake in 2019 (50% of current drug users started treatment) [58,59], followed by Australia, where 41% of active drug users started treatment in 2018–2019 [58,60,61]. 

## 5. Conclusions

Much work needs to be done to eliminate HCV infection globally. Of the 45 high-income countries, only 11 are on track to meet the WHO’s elimination targets by 2030 [62]. New strategies and targeted interventions are necessary to enhance patient engagement. Here, we try to show that each step taken by medical professionals toward the high-risk PWID subgroup of potential patients is helpful.

Our off-site service allowed 168 people to access testing services. With a low-barrier approach to working with PWIDs and with the respect for their specific needs and barriers, it is possible to establish therapeutic relationships and offer testing and DAA treatment when medically appropriate. The fact that the outreach service operates in an area in which many PWIDs congregate helps them to come and participate. We offer a shortcut for PWIDs to become involved in the professional healthcare system. In addition, the fact that the “Ambulance” outreach program in Remedis has a direct link with the Center for Treatment of Viral Hepatitis of Remedis supports patients’ confidence and decision to participate in HCV treatment regardless of their current social situation. This model is promising and could become one piece of the puzzle of eliminating HCV infection globally.

## Figures and Tables

**Table 1:** Basic principles of the Comprehensive Care Program (CCP) for patients with addiction comorbidity [16].

Basic Principles Comprehensive Care Program for Patients with Addiction Comorbidity
▪ Access to health care and preventive care with no restrictions ▪ Everything is under one roof—easy access to health care ▪ Easy-to-use system for making appointments ▪ Working hours adjusted to the patients’ needs—time available health care ▪ Interconnected services—follow-up care ▪ Individual diagnostic and treatment plan for each patient ▪ Friendly, proactive approach to patients at all levels of contact and care ▪ Dissemination of information by patients themselves (peer to peer) ▪ Using feedback from patients to evaluate and further improve CCP’s services and make them even more available.

**Table 2:** Serological results. N/A—not available (patients were not serologically tested due to a small amount of serum sample).

Clients	Results	Anti HAV-IgM	Anti HAV-IgG	HBsAg	Anti-HBc	Anti-HBs	Anti-HCV	Anti-HIV
n = 168	positive	0	96	0	69	89	133	0
	negative	161	66	164	94	76	32	168
	N/A	7	6	4	5	3	3	0
Total		168	168	168	168	168	168	168
Female	positive	0	30	0	17	33	40	0
	negative	51	21	50	33	18	10	52
	N/A	1	1	2	2	1	2	0
Total		52	52	52	52	52	52	52
Male	positive	0	66	0	52	56	93	0
	negative	110	45	114	61	58	22	116
	N/A	6	5	2	3	2	1	0
Total		116	116	116	116	116	116	116

**Table 3:** PWID clients recruited in the outreach program who successfully started DAA treatment. PIB—pibrentasvir, GLE—glecaprevir, GZR—grazoprevir, EBR—elbasvir, SOF—sofosbuvir, VEL—vepatasvir, SVR—sustained virological response defined as undetectable HCV RNA 12 weeks after treatment completion, N/A—data not available, Ø—average; Office—doctor’s office in the Remedis outpatient clinic; O—other healthcare facility; * treatment realized in another healthcare facility (IKEM Prague); ** HCV reinfection confirmed by PCR.

Patient’s Number	Gender Male/Female	Age	1st Contact with Remedis	1st Contact in Ambulance (MM/YYYY)	HCV Genotype	FIBROSCAN (kPa)	HCV Treatment Started (MM/YYYY)	Time from 1st Contact till Treatment (M)	Where Treated	Treatment Regimen	SVR Yes/No
1	F	36	Ambulance	12/2019	1a	5.8	7/2020	7	PRISON	PIB/GLE	YES
2	F	33	OFFICE	10/2019	1a	4.3	2/2020	4	OFFICE	PIB/GLE	YES
3	F	51	Ambulance	2/2020	1b	9.9	10/2020	8	OFFICE	GZR/EBR	YES
4	M	45	Ambulance	10/2019	3	10.1	9/2020	11	OFFICE	PIB/GLE	N/A
5	M	59	OFFICE	7/2019	1a	12.8	3/2021	20	PRISON	PIB/GLE	N/A
6	F	37	OFFICE	8/2020	3	8.5	3/2021	7	OFFICE	PIB/GLE	YES
7	M	36	Ambulance	1/2020	3	7.1	2/2020	1	OFFICE	PIB/GLE	NO **
8	M	38	PRISON	1/2020	1a	7.8	1/2020	0	OFFICE	PIB/GLE	N/A
9	M	50	OFFICE	11/2019	3	0	1/2020	0	O *	N/A	N/A **
10	F	26	Ambulance	2/2020	3	6.2	5/2020	3	OFFICE	SOF/VEL	YES
11	M	34	Ambulance	1/2020	1a	0	6/2020	0	O *	N/A	N/A
12	F	39	OFFICE	7/2019	3	7.3	10/2019	3	OFFICE	PIB/GLE	YES
13	M	37	OFFICE	2/2020	1a	11.3	4/2021	14	OFFICE	PIB/GLE	YES
14	M	38	OFFICE	9/2019	3	43.7	10/2019	1	OFFICE	SOF/VEL	YES
15	F	40	Ambulance	10/2019	1a/1b	7	6/2021	20	PRISON	PIB/GLE	YES
16	F	28	Ambulance	1/2020	3/1a	4.7	4/2020	3	OFFICE	PIB/GLE	YES
17	M	35	Ambulance	3/2021	3	8.8	4/2021	1	OFFICE	SOF/VEL	N/A
18	F	41	OFFICE	11/2019	3	8	7/2020	8	PRISON	PIB/GLE	YES
19	F	40	OFFICE	7/2019	1a	33.5	10/2020	15	OFFICE	SOF/VEL	YES
20	M	37	OFFICE	11/2019	1a	6.4	2/2020	3	OFFICE	PIB/GLE	YES
21	M	43	Ambulance	10/2019	1a	6.1	11/2019	1	OFFICE	PIB/GLE	YES
22	F	37	OFFICE	7/2019	1b	4	5/2020	10	PRISON	GZR/EBR	YES
23	M	43	OFFICE	10/2019	3	6.6	2/2020	4	OFFICE	PIB/GLE	YES
24	M	38	OFFICE	10/2020	1b	5.1	12/2020	2	OFFICE	PIB/GLE	YES
		Ø 39.2				Ø 9.4		Ø 6.08			

## Data Availability

Data sharing not applicable.
